# Supplementary material for: More Shots on Goal: Heterologous Influenza Prime-Boost Vaccination Decreases the Likelihood of a Negligible Antibody Response to Current or Historical Variants
Source: medRxiv. 2026 Oct 30:2026.10.28.26366744. Preprint. [Version 1] doi: 10.64898/2026.10.28.26366744 (PMC13734158; doi:10.64898/2026.10.28.26366744)
Supplement: Supplementary file 1 [file NIHPP2026.10.28.26366744v1-supplement-1.pdf]

medRxiv preprint doi: https://doi.org/10.64898/2026.10.28.26366744; this version posted October 30, 2026. The copyright holder for this preprint (which was not certified by peer review) is the author/funder, who has granted medRxiv a license to display the preprint in perpetuity.
It is made available under a CC-BY 4.0 International license .

Supplementary Figures and Tables

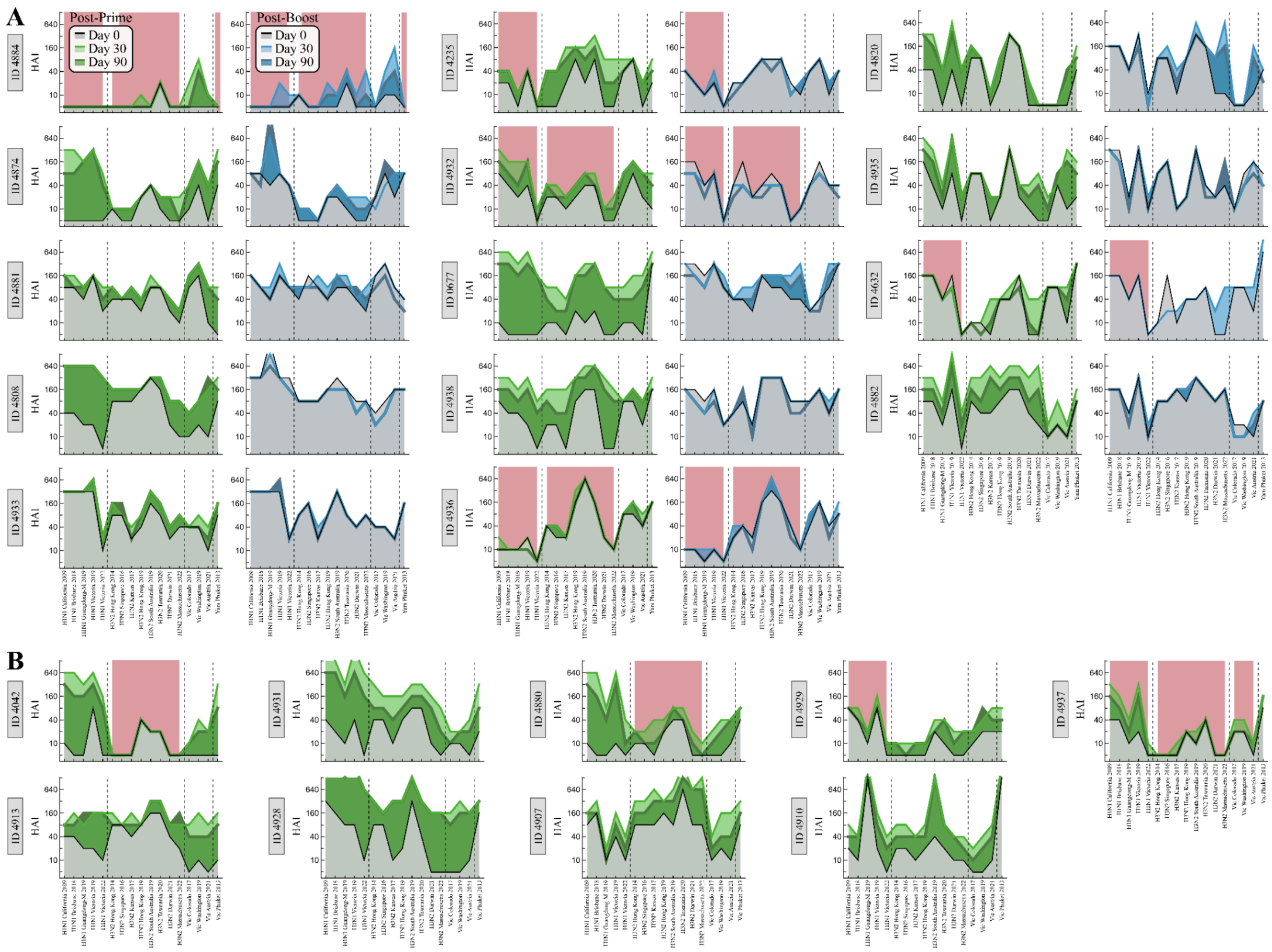

Figure S1. Individual antibody landscapes for the LJI cohort. Titers at all time points from subjects that (A) received the prime and booster vaccines or (B) only received the priming vaccine. In each panel, subjects are sorted from least-to-greatest day 0 GMT across all variants. H1N1, H3N2, B/Victoria, or B/Yamagata strains have a red highlight for subjects with HAI titers≤10 for all time points for that vaccine strain (with either H3N2 vaccine strain counting).

medRxiv preprint doi: https://doi.org/10.64898/2026.10.28.26366744; this version posted October 30, 2026. The copyright holder for this preprint (which was not certified by peer review) is the author/funder, who has granted medRxiv a license to display the preprint in perpetuity.
It is made available under a CC-BY 4.0 International license .

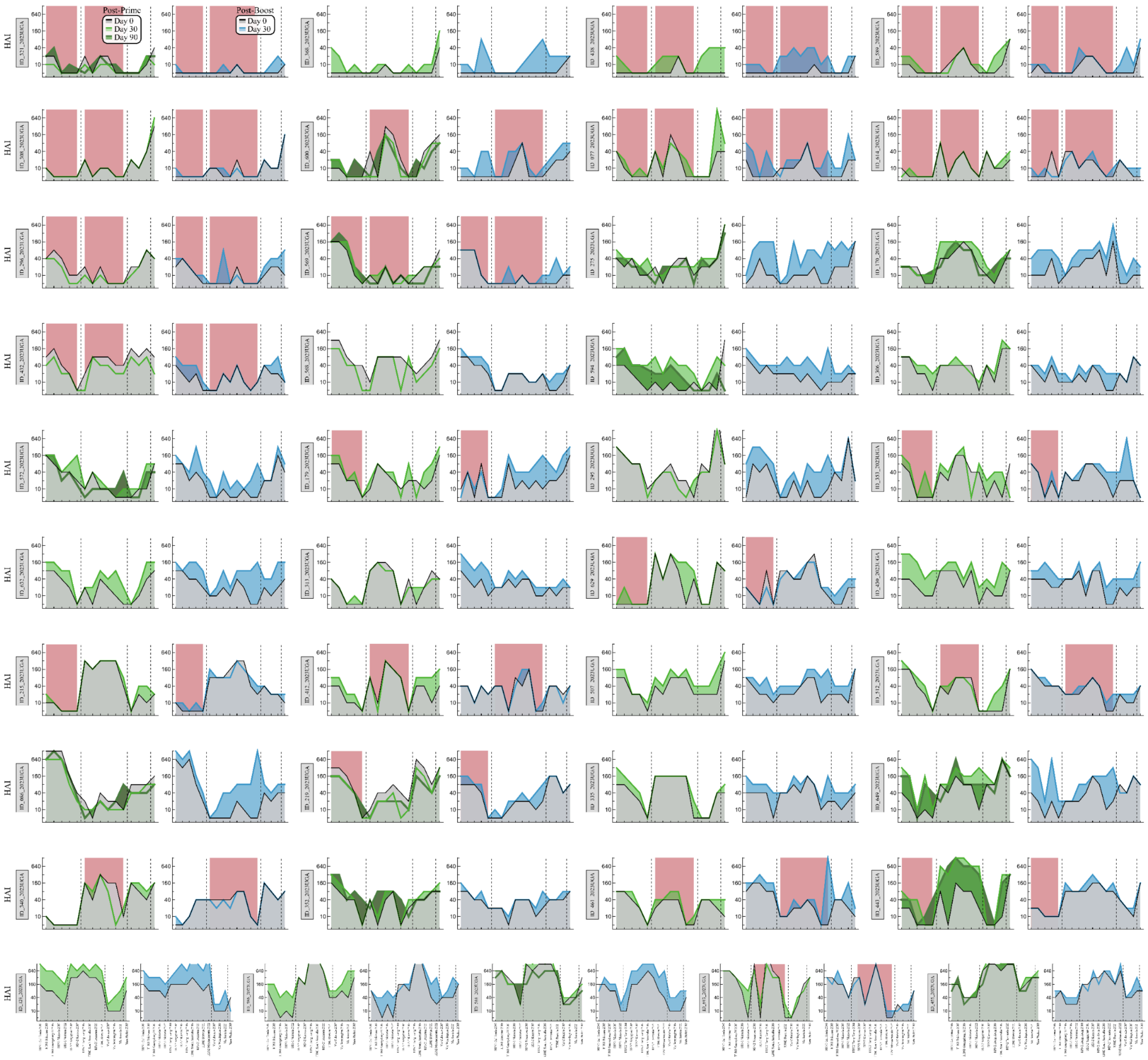

Figure S2. Individual antibody landscapes for the UGA cohort. Titers at all time points from UGA subjects receiving a vaccine in the fall of 2023 and the fall of 2024. Subjects are sorted from least-to-greatest day 0 GMT across all variants. The 2023 virus panel did not include H3N2 Hong Kong 2014 or Massachusetts 2022, and H1N1 Victoria 2022 was not measured at day 90. H1N1, H3N2, B/Victoria, or B/Yamagata strains have a red highlight for subjects with HAI titers≤10 for all time points for that vaccine strain (with either H3N2 vaccine strain counting).

medRxiv preprint doi: https://doi.org/10.64898/2026.10.28.26366744; this version posted October 30, 2026. The copyright holder for this preprint (which was not certified by peer review) is the author/funder, who has granted medRxiv a license to display the preprint in perpetuity.
It is made available under a CC-BY 4.0 International license .

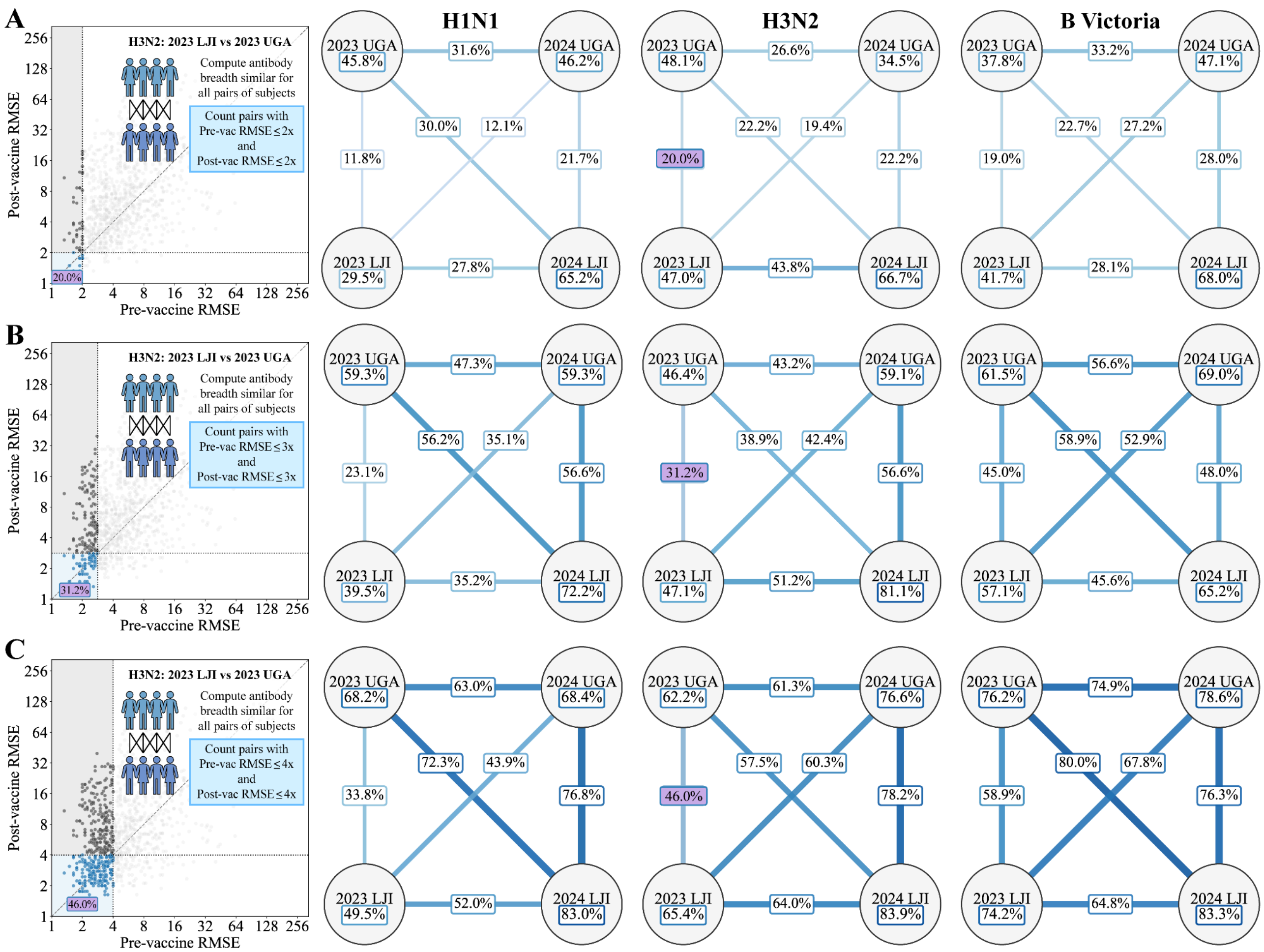

Figure S3. RMSE-based matching of antibody landscapes between LJI and UGA study cohorts. Pre- and post-vaccination RMSE distribution for all 2023 UGA and 2023 LJI subject pairs against H3N2 variants, with the purple region indicating pairs meeting the similarity threshold of (A) RMSE≤2x, (B) ≤3x, and (C) ≤4x. Each threshold's corresponding cohort similarity networks for H1N1, H3N2, and B/Victoria are shown to the left. Connection labels indicate the fraction of subject pairs exhibiting pre- and post-vaccine RMSE less than or equal to each threshold across internal cohort comparisons and cross-cohort alignments. B/Yamagata was excluded because only a single variant was measured from that lineage.

Table S1. Statistical significance of HAI titer increases at each time point compared to day 0, day 120, or between the 2023-2024 H3N2 vaccine strains in the LJI cohort. *p*-values were based on one-sided t-test with Holm correction applied for each influenza vaccine strain. When comparing the 2023-2024 H3N2 vaccine strains, the strain in the most recently administered vaccine was expected to have the higher titer (*i.e.*, H3N2 A/Darwin/9/2021 at days 0, 30, and 90; H3N2 A/Massachusetts/18/2022 at days 120, 150, and 210).

Influenza Vaccine Strains in 2023 and 2024	Day 30 vs Day 0	Day 90 vs Day 0	Day 120 vs Day 0	Day 150 vs Day 0	Day 210 vs Day 0
H1N1 A/Victoria/4897/2022	<0.001	<0.001	0.014	0.010	0.014

medRxiv preprint doi: https://doi.org/10.64898/2026.10.28.26366744; this version posted October 30, 2026. The copyright holder for this preprint (which was not certified by peer review) is the author/funder, who has granted medRxiv a license to display the preprint in perpetuity.
It is made available under a CC-BY 4.0 International license .

H3N2 A/Darwin/9/2021	<0.001	0.001	0.015	0.001	0.007
H3N2 A/Massachusetts/18/2022	<0.001	0.002	0.006	<0.001	<0.001
Victoria B/Austria/1359417/2021	<0.001	<0.001	<0.001	<0.001	<0.001
Yamagata B/Phuket/3073/2013	0.003	0.070	0.312	0.312	0.352

Influenza Vaccine Strains in 2023 and 2024	Day 150 vs Day 120	Day 210 vs Day 120
H1N1 A/Victoria/4897/2022	0.841	0.841
H3N2 A/Darwin/9/2021	0.321	0.321
H3N2 A/Massachusetts/18/2022	0.031	0.071
Victoria B/Austria/1359417/2021	0.017	0.121
Yamagata B/Phuket/3073/2013	0.916	0.916

Influenza Vaccine Strains	Day 0	Day 30	Day 90	Day 120	Day 150	Day 180
H3N2 A/Darwin/9/2021 vs H3N2 A/Massachusetts/18/2022	0.913	0.913	0.913	0.913	0.396	0.708

Table S2. Statistical significance of HAI titer increases at each time point compared to day 0, day 365, or between the 2023-2024 H3N2 vaccine strains in the UGA cohort. *p*-values based on one sided t-test, with Holm correction applied for each influenza vaccine strain. Unlike in the LJI cohort, day 90 titers were not measured for H1N1, and since the H3N2 A/Massachusetts/18/2022 was not measured at day 0, 30, or 90. When comparing the 2023-2024 H3N2 vaccine strains at days 365 and 395, H3N2 A/Massachusetts/18/2022 was expected to have the higher titers.

Influenza Vaccine Strains in 2023 and 2024	Day 30 vs Day 0	Day 90 vs Day 0	Day 365 vs Day 0	Day 395 vs Day 0
H1N1 A/Victoria/4897/2022	0.040	N/A	0.019	<0.001
H3N2 A/Darwin/9/2021	0.071	0.191	0.898	0.071
Victoria B/Austria/1359417/2021	0.006	0.266	0.964	0.266
Yamagata B/Phuket/3073/2013	1	1	1	1

medRxiv preprint doi: https://doi.org/10.64898/2026.10.28.26366744; this version posted October 30, 2026. The copyright holder for this preprint (which was not certified by peer review) is the author/funder, who has granted medRxiv a license to display the preprint in perpetuity.
It is made available under a CC-BY 4.0 International license .

Influenza Vaccine Strains in 2023 and 2024	Day 395 vs Day 365
H1N1 A/Victoria/4897/2022	<0.001
H3N2 A/Darwin/9/2021	<0.001
H3N2 A/Massachusetts/18/2022	<0.001
Victoria B/Austria/1359417/2021	<0.001
Yamagata B/Phuket/3073/2013	0.074

Influenza Vaccine Strains	Day 365	Day 395
H3N2 A/Darwin/9/2021 vs H3N2 A/Massachusetts/18/2022	0.283	0.011
